# Associations Between School Characteristics and Classroom Radon Concentrations in Utah’s Public Schools: A Project Completed by University Environmental Health Students

**DOI:** 10.3390/ijerph17165839

**Published:** 2020-08-12

**Authors:** Elizabeth A. Davis, Judy Y. Ou, Cheyenne Chausow, Marco A. Verdeja, Eleanor Divver, James D. Johnston, John D. Beard

**Affiliations:** 1 Department of Public Health, Brigham Young University, Provo, UT 84602, USA; eapowell@uci.edu (E.A.D.); c.chausow17@gmail.com (C.C.); marcoverdeja@hotmail.com (M.A.V.); james_johnston@byu.edu (J.D.J.); 2 Cancer Control and Population Sciences, Huntsman Cancer Institute at the University of Utah School of Medicine, Salt Lake City, UT 84112, USA; Judy.Ou@hci.utah.edu; 3 Radon Program, Utah Department of Environmental Quality, Salt Lake City, UT 84116, USA; edivver@utah.gov

**Keywords:** environmental, exposure assessment, occupational, radon, school

## Abstract

Radon (²²²Rn), a radioactive gas, is the second leading cause of lung cancer deaths in the U.S. Classroom radon concentrations in public schools in our target area had never been measured or had not been measured in many years. We had university students, primarily enrolled in environmental health courses, measure radon concentrations in 2289 classrooms in 66 of Utah’s public schools and identify school characteristics associated with classroom radon concentrations. The geometric mean (GM) classroom radon concentration was 31.39 (95% confidence interval (CI): 27.16, 36.28) Bq/m^3^ (GM: 0.85; 95% CI: 0.72, 0.98 pCi/L). Thirty-seven (2%) classrooms in 13 (20%) schools had radon concentrations at or above the U.S. Environmental Protection Agency’s (EPA) recommended action level of 148 Bq/m^3^ (4.0 pCi/L). Number of classrooms had a u-shaped association with classroom radon concentrations. The year the heating, ventilation, and air conditioning (HVAC) system was installed was inversely associated with having classroom radon concentrations at or above the EPA’s recommended action level. Number of classrooms and number of students had u-shaped associations with having classroom radon concentrations at or above the EPA’s recommended action level. Classroom radon concentrations decreased when schools’ HVAC systems were on. Replacing HVAC systems and turning/keeping them on may be effective radon mitigation strategies to prevent radon-associated lung cancer, especially for small and large schools.

## 1. Introduction

Radon (²²²Rn) is a radioactive gas produced by the breakdown of uranium and is undetectable by the senses [1]. When released by rocks or soil [2], radon may enter buildings through cracks in building foundations and get trapped in enclosed spaces. This increases air concentrations of radon and exposes people to inhalable radon, which is typically the public’s largest source of background radiation exposure [1]. As people breathe in radon gas, radioactive particles get trapped in their lungs, thereby increasing their risk of lung cancer [1]. Radon exposure is the leading cause of lung cancer among nonsmokers and the second leading cause of lung cancer among smokers. Approximately one in 143 nonsmokers, and one in 16 smokers, exposed to 148 becquerels of radon per cubic meter of air (Bq/m^3^; 4.0 picocuries per liter of air [pCi/L]) for a lifetime will develop lung cancer [3]. Children exposed to radon are twice as likely compared to adults to develop lung cancer when exposed to the same concentrations [3]. Children’s smaller lungs, faster breathing rates, and lower proximity to the ground results in higher levels of radon exposure and, therefore, a higher risk of developing lung cancer [3]. More than 20,000 lung cancer deaths in the U.S. each year are attributed to radon [1]. Although the state of Utah has the lowest smoking rate in the United States [4], lung cancer is the number one cause of cancer mortality in Utah [5].

School-age children and school employees, including teachers, administrators, maintenance workers, and staff, spend a significant amount of time in school buildings. The U.S. Environmental Protection Agency (EPA) advises school buildings be tested for radon at least once every five years [6]. Testing a building for radon is the only way to know if the indoor radon concentration is below or above the EPA’s recommended action level of 148 Bq/m^3^ (4.0 pCi/L; i.e., the level at or above which EPA recommends mitigation to lower radon concentrations). There are many factors that may contribute to elevated radon concentrations in schools, including radon in the soil near a school building and the permeability of the soil, the way the school buildings were constructed, and the type, use, and upkeep of the heating, ventilation, and air conditioning (HVAC) system in the school [6]. The school’s HVAC system may play a significant role in indoor radon concentrations by increasing or decreasing air exchange rates, or by causing fluctuations in indoor pressure relative to below surface areas of the school, such as basements, crawl spaces, and utility tunnels [6].

In Utah, 30% of homes tested for radon have concentrations at or above the EPA’s recommended action level of 148 Bq/m^3^ (4.0 pCi/L) [7]. The average radon concentration in Utah’s homes is 196.10 Bq/m^3^ (5.3 pCi/L), with a maximum of 24,568 Bq/m^3^ (664 pCi/L) [8]. There is little knowledge regarding indoor radon concentrations in Utah’s public schools due to minimal radon testing (no laws require radon testing in Utah’s public schools), a long time since previous testing, and the construction of many new public schools over the past couple of decades to accommodate Utah’s growing population. The current gap in knowledge regarding radon concentrations in Utah’s public schools is potentially problematic due to the serious health implications (i.e., lung cancer) of radon for those exposed, especially children. To address this gap and to increase the quantity of data regarding indoor radon concentrations in Utah’s public schools, Brigham Young University (BYU) collaborated with the Utah Department of Environmental Quality (UDEQ), the Huntsman Cancer Institute at the University of Utah School of Medicine, and two of Utah’s public school districts to measure radon concentrations in schools. The first objective of our study was to measure radon concentrations in each ground or basement level classroom of each school of the partnering school districts and encourage any schools that had classroom radon concentrations at or above the EPA’s recommended action level to form a radon mitigation plan. The second objective of our study was to estimate associations between school characteristics and classroom radon concentrations to identify determinants of higher or lower radon concentrations in public schools.

## 2. Materials and Methods

### 2.1. Study Design and Population

**Figure 1:**
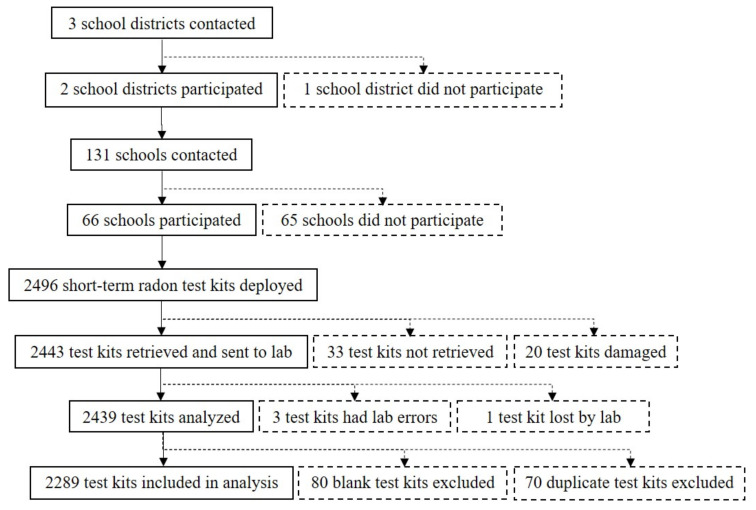
Flow diagram depicting short-term radon test kits analyzed by schools and school districts that participated in a cross-sectional study of radon concentrations in Utah’s public schools. Solid boxes or lines depict school districts, schools, or test kits that progressed past each step shown; dashed boxes or lines depict school districts, schools, or test kits excluded after each step shown. The 33 test kits that were not retrieved were in locked rooms, had been discarded, or could not be found.

During January and February 2019, we conducted a cross-sectional study by testing 2289 ground or basement level classrooms in 66 public schools in Utah. EPA recommends short-term radon tests be conducted during the winter when radon concentrations are typically the highest [9]. We submitted an application to BYU’s Institutional Review Board (IRB) who determined that our study did not meet the definition of human subjects’ research and thus did not require IRB approval. We also submitted a request to perform research in Utah’s school districts via BYU’s David O. McKay School of Education, which has a long-standing relationship with many of Utah’s school districts. We recruited participating schools through in-person meetings with administrators at three school districts (Figure 1). Two districts agreed to participate. With the districts’ permission, we contacted and recruited individual school principals and head custodians at 131 schools via email and telephone calls. We followed up with schools up to three times until they agreed or declined to participate. We scheduled days and times to test the 66 participating schools and administer a survey regarding school characteristics that may be associated with classroom radon concentrations. Study personnel consisted of 100 individuals, including faculty members and 94 BYU undergraduate and graduate students, who assisted with testing schools for radon. Most students were enrolled in environmental health courses and assisted with testing to fulfill requirements for experiential/service learning assignments. We deployed 2496 short-term radon test kits, retrieved and mailed 2443 test kits to the lab for analysis, received results from the lab for 2439 test kits, and included 2289 test kits/classrooms in our analysis after removing blank and duplicate test kits (Figure 1).

### 2.2. Exposure Assessment

To fulfill the first objective of our study, we tested schools using short-term radon test kits, which use activated charcoal for the adsorption of radon gaseous atoms [10]. Activated charcoal is a widely accepted method used in short-term radon test kits to determine radon concentrations. The short-term radon test kits we used were purchased by the UDEQ. This type of test kit is used by local health departments and homeowners. Alpha Energy Labs (Carrollton, TX, USA) produces, mails, and analyzes the short-term radon test kits we used to test the schools. The laboratory is certified and meets and exceeds the EPA’s standards for measuring short-term radon test kits [11].

Study personnel arrived at each school either before or after normal school hours, depending on the schools’ preferences, to deploy and retrieve the short-term radon test kits. Study personnel checked in with the office staff upon arrival at the schools and checked out upon leaving. Study personnel worked in teams of two to conduct the testing with one person deploying/retrieving the test kits and the other recording the locations of the test kits (e.g., classroom number and location in the classroom such as on a bookshelf, filing cabinet, etc.). Two study teams (i.e., four people total) tested larger schools. We gave study personnel the instructions provided in the EPA’s guidelines for deploying the short-term radon test kits (Supplementary Material, p. 2) [10].

Study personnel retrieved the short-term radon test kits 48–72 h after they were deployed to each classroom. We mailed the test kits to Alpha Energy Labs for analysis. We emailed results to the principals and head custodians of each school and/or administrators of each school district.

We encouraged schools that had at least one classroom that tested at or above the EPA’s recommended action level of 148 Bq/m^3^ (4.0 pCi/L) [12] to contact study staff for assistance with follow-up testing. To confirm the elevated radon concentrations at schools, we used three Model 1028 Continuous Radon Monitors (CRM) (SunRadon, LLC, Melbourne, FL, USA) to conduct follow-up testing during February 2019 in 14 classrooms in two schools that tested at or above the EPA’s recommended action level. The CRMs were calibrated on July 6, 2018, by Bowser-Morner, Inc. (Dayton, OH, USA). The CRMs gave hourly radon measurements so we could evaluate temporal patterns in radon concentrations. We collected between 48 and 90 h of CRM data for each of the 14 classrooms and one office (in a library) nearby (i.e., we used CRMs to test 15 rooms total).

According to the manufacturer’s instructions, CRMs require operation by trained, state-certified testers. Accordingly, all CRM measurements in this study were collected by a trained and certified staff member. We emailed results of the CRM testing to the principals and head custodians of each school and/or administrators of each school district. We encouraged schools to contact study staff for assistance with mitigation.

To fulfill the second objective of our study, we developed a 20 question survey regarding school characteristics that may be associated with classroom radon concentrations in schools (Supplementary Material, p. 4). The survey included questions about the following topics: the school’s HVAC system (e.g., type of HVAC system, hours of operation, age of HVAC system, etc.), school age, school structure (e.g., number of stories, presence or absence of a basement, number of classrooms, etc.), and previous radon mitigation. We administered the survey to school principals and head custodians at participating schools during the weeks we tested their schools for radon. We emailed a link to an online Qualtrics (Qualtrics, Provo, UT, USA) version of the survey and study personnel provided a paper copy of the survey when they deployed the short-term radon test kits at the schools. Study personnel retrieved the paper copy of the survey, if it had been completed, when they retrieved the short-term radon test kits from the schools. Later, we recontacted schools via email and/or telephone to clarify answers to the survey, complete questions that were originally unanswered, or obtain answers if they had not yet completed the survey. Of the 66 participating schools, 65 schools completed the survey and one school declined.

### 2.3. Statistical Analyses

We completed our analyses utilizing SAS version 9.4 (SAS Institute, Inc., Cary, NC, USA). We calculated mean, standard deviation, minimum, first quartile, median, third quartile, and maximum values for continuous school characteristics and radon variables. We calculated frequencies and percentages for categorical school characteristics and radon variables.

We calculated the geometric mean (GM), and 95% confidence interval (CI), classroom radon concentration using an intercept only Tobit mixed regression model that had natural logarithm transformed radon concentrations as the dependent variable to account for radon concentrations being below detection limits (35% of classroom radon concentrations were below detection limits, which ranged from 11.1 to 55.5 Bq/m^3^ [0.3–1.5 pCi/L]) and correlations between radon concentrations in classrooms in the same schools. We calculated the GM because the distribution of classroom radon concentrations was right skewed. We used PROC NLMIXED for this analysis, as used in a paper by Jin et al. [13], which included SAS code that we modified.

We used simple Tobit mixed regression models that had natural logarithm transformed classroom radon concentrations as the dependent variable to estimate GMs or GM ratios, 95% CIs, and *p*-values for unadjusted bivariate associations between school characteristics and classroom radon concentrations. We calculated GMs for categorical school characteristics and GM ratios for continuous school characteristics. When appropriate, we evaluated pairwise differences in GM classroom radon concentrations among categories of school characteristics and used the Bonferroni method to adjust the significance level for multiple comparisons.

We used simple unconditional logistic regression models with generalized estimating equations to estimate odds ratios (ORs) and 95% CIs for unadjusted bivariate associations between school characteristics and (1) having a classroom radon concentration at or above the EPA’s recommended action level (i.e., 148 Bq/m^3^ [4.0 pCi/L]; [12]) and (2) having a classroom radon concentration above detection limits. We used generalized estimating equations to account for correlations between measurements in classrooms in the same schools. We used the quasilikelihood under the independence model criterion (QIC) to determine the structure of the working correlation matrix (i.e., we used the working correlation matrix that had the lowest QIC) [14]. We used an exchangeable working correlation matrix for analyses of having a classroom radon concentration at or above the EPA’s recommended action level and an independent working correlation matrix for analyses of having a classroom radon concentration above detection limits.

We considered several versions of the school characteristics variables (e.g., continuous, categorical based on quintiles, categorical based on 10 year increments, categorical based on five year increments, etc.). We used the Akaike Information Criterion (AIC) for the Tobit mixed regression models [15,16] and the QIC for the unconditional logistic regression models with generalized estimating equations [14] to determine the versions of the school characteristics variables that we used for the analyses (i.e., we used the versions that had the lowest AIC/QIC). For categorical versions of the school characteristics variables included in unconditional logistic regression models with generalized estimating equations, we conducted linear trend tests by including within-category medians, calculated using all classrooms, as continuous variables in the model [17]. When necessary, we scaled GM ratios, ORs, and 95% CIs (e.g., to a 10 year increase) to make them easier to interpret.

We used Moran’s I to conduct a test for spatial autocorrelation of the natural logarithm of the GM classroom radon concentrations of each school to ensure that our regression model findings were a product of the associations between school characteristics and classroom radon concentrations rather than the schools’ spatial proximity to each other.

SAS does not currently have the option to conduct stepwise variable selection for Tobit mixed regression models or unconditional logistic regression models with generalized estimating equations. Therefore, we attempted to conduct manual stepwise variable selection, but experienced errors in estimation, particularly of covariance matrices and *p*-values, which rendered this approach unfeasible. However, we were able to estimate some Tobit mixed regression models and unconditional logistic regression models with generalized estimating equations that included more than one school characteristic as independent variables.

We plotted the data collected using the CRMs via line graphs that showed classroom and mean radon concentrations over time.

## 3. Results

Forty-six (70%) schools were elementary schools, 12 (18%) were junior high or middle schools, and eight (12%) were high schools (Table 1). The median age of the schools we tested was 23.0 years old, the median year of HVAC installation was 2002, and the HVAC system had been maintained during 2019 for 24 (39%) schools. Forty-five (71%) schools had multizone HVAC systems, six (10%) had either a single-zone or a hydronic system, six (10%) had a variable air volume system, and six (10%) had HVAC systems that were combinations of different types. The median time the HVAC system was turned on each day was 06:15:00 and the median time the HVAC system was turned off was 16:00:00 for a median number of hours the HVAC system was on/running of 9.90 h. All schools had their HVAC systems on/running during school hours. Twenty (31%) schools had basements, of which five (26%) were finished, 14 (23%) schools had a crawlspace or uncovered dirt floor, and 48 (75%) schools had only one level/story. The median number of classrooms in the schools was 38, and the median number of students was 819. Only 15 (31%) schools were reported to have been built using radon-resistant new construction.

Of the 2289 classrooms we tested, 798 (35%) had radon concentrations that were below detection limits (Table 2). The GM radon concentration of all the classrooms we tested was 31.39 (95% CI: 27.16, 36.28) Bq/m^3^ (GM: 0.85; 95% CI: 0.73, 0.98 pCi/L), and the maximum concentration was 673.40 Bq/m^3^ (18.20 pCi/L). Thirty-seven (2%) classrooms in 13 (20%) different schools had radon concentrations at or above the EPA’s recommended action level of 148 Bq/m^3^ (4.0 pCi/L).

Number of classrooms in the schools was associated with classroom radon concentrations (*p* = 0.03) (Table 3). Small (19–30 classrooms: GM: 42.60; 95% CI: 30.95, 58.64 Bq/m^3^; GM: 1.15; 95% CI: 0.84, 1.58 pCi/L) and large (81–148 classrooms: GM: 58.59; 95% CI: 32.03, 107.18 Bq/m^3^; GM: 1.58; 95% CI: 0.87, 2.90 pCi/L) schools had higher classroom radon concentrations than medium schools (e.g., 41–50 classrooms: GM: 23.02; 95% CI: 15.84, 33.46 Bq/m^3^; GM: 0.62; 95% CI: 0.43, 0.90 pCi/L) and the following pairwise comparisons of GM classroom radon concentrations among classroom categories were significantly different: 19–30 vs. 41–50: *p* = 0.02; 19–30 vs. 51–60: *p* = 0.02; 31–40 vs. 81–148: *p* = 0.03; 41–50 vs. 81–148: *p* = 0.01; and 51–60 vs. 81–148: *p* = 0.01. Using a Bonferroni correction, however, none of these differences were significant, as that would have required a *p*-value lower than 0.00238. No other school characteristic had a significant relationship with classroom radon concentrations.

For a ten-year increase in school age, the odds of having a classroom radon concentration at or above the EPA’s recommended action level increased nonsignificantly by 20% (95% CI: −5%, 51%) (Table 4). The odds of having a classroom radon concentration at or above the EPA’s recommended action level decreased by 28% (95% CI: −1%, −47%) for a 10 year increase in the year the HVAC system was installed (i.e., the odds were lower if the HVAC system was installed more recently). Classrooms in schools that turned off their HVAC systems between 16:00:01 and 17:00:00 had 6.85 (95% CI: 1.38, 34.14) times the odds of having a radon concentration at or above the EPA’s recommended action level compared to classrooms in schools that turned off their HVAC systems between 15:00:00 and 16:00:00. Using 31–70 classrooms as the reference, classrooms in schools with 19–31 and 71–148 classrooms had 19.40 (95% CI: 4.59, 81.99) and 6.53 (95% CI: 2.04, 20.84), respectively, times the odds of having a radon concentration at or above the EPA’s recommended action level. The linear trend odds ratio was a nonsignificant 0.92 (95% CI: 0.53, 1.60) for a 10 classroom increase, which, considered together with the categorical odds ratios, suggests a nonlinear relationship between number of classrooms and having a classroom radon concentration at or above the EPA’s recommended action level. Similarly, using 651–1500 students as the reference, classrooms in schools with 182–650 and 1501–3300 students had 9.32 (95% CI: 2.23, 38.99) and 4.78 (95% CI: 1.33, 17.24), respectively, times the odds of having a radon concentration at or above the EPA’s recommended action level. The linear trend odds ratio was a nonsignificant 0.95 (95% CI: 0.75, 1.19) for a 100 student increase, which, considered together with the categorical odds ratios, suggests a nonlinear relationship between number of students and having a classroom radon concentration at or above the EPA’s recommended action level.

Although there were some significant associations between having a classroom radon concentration above detection limits and certain categories of time the HVAC system was turned on each day, time the HVAC system was turned off each day, and number of classrooms, no clear patterns were found among associations between school characteristics and having a classroom radon concentration above detection limits (Supplementary Material, Table S1). Classrooms in schools that had been mitigated for radon previously had 2.21 (95% CI: 0.98, 4.98) times the odds of having a radon concentration above detection limits than classrooms in schools that had not been mitigated for radon previously.

There was no evidence of spatial autocorrelation of the natural logarithm of the GM classroom radon concentrations of each school (*p* = 0.98).

Although we experienced errors in estimation for some Tobit mixed regression models and unconditional logistic regression models with generalized estimating equations that included more than one school characteristic as independent variables, number of classrooms in the schools remained statistically significantly associated with classroom radon concentrations after adjusting for every other school characteristic one-at-a-time (not shown). Similarly, number of classrooms in the schools remained statistically significantly associated with having a classroom radon concentration at or above the EPA’s recommended action level after adjusting for every other school characteristic one-at-at-time except for school grade and basement present (not shown). When adjusting for school grade, the *p*-value for number of classrooms in the schools was 0.06 and the *p*-value for school grade was 0.41. When adjusting for basement present, the *p*-value for number of classrooms in the schools was 0.05 and the *p-*value for basement present was 0.67.

**Figure 2:**
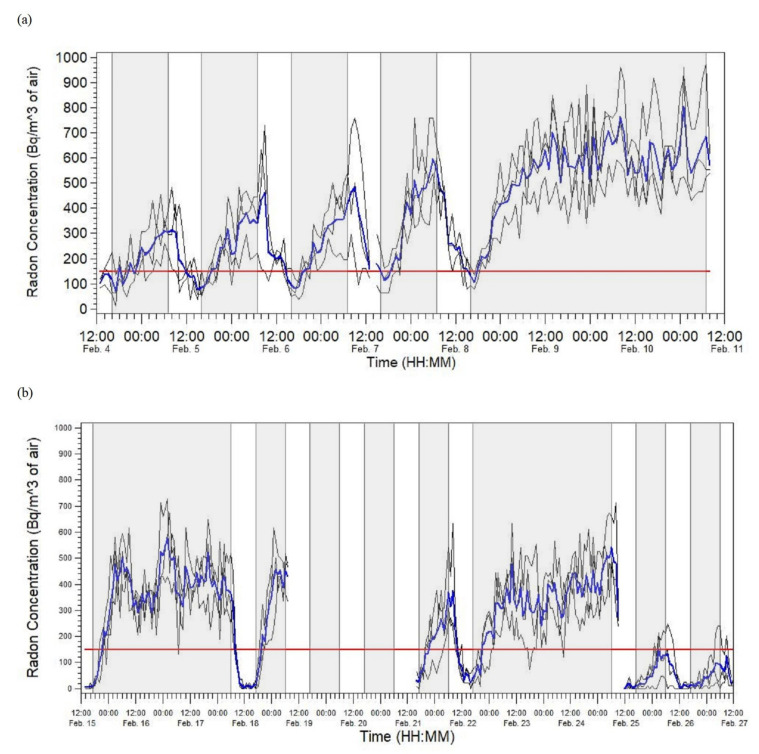
Radon concentrations measured by CRMs in two schools that collectively had 14 classrooms that had short-term radon concentrations at or above the EPA’s recommended action level for radon (i.e., 148 Bq/m^3^ [4.0 pCi/L]; [12]). (**a**) Radon concentrations measured by CRMs in five classrooms and one office (in the library) at the first school. Three classrooms were monitored from February 4 to 7, 2019, and two classrooms and one office were monitored from February 7 to 11, 2019. (**b**) Radon concentrations measured by CRMs in nine classrooms at the second school. Three classrooms were monitored from February 15 to 19, 2019, three classrooms were monitored from February 21 to 25, 2019, and three classrooms were monitored from February 25 to 27, 2019. In each plot, individual classroom radon concentrations are shown via black lines, mean radon concentrations are shown via blue lines, the EPA’s recommended action level for radon is shown via red lines, the time period the HVAC system was off is shaded in gray, and the time period the HVAC system was on is unshaded. Abbreviations: Bq/m^3^, becquerels per cubic meter of air; CRM, continuous radon monitor; HVAC, heating, ventilation, and air conditioning; pCi/L, picocuries per liter of air; EPA, U.S. Environmental Protection Agency.

The data collected via the CRMs shown in panels (a) and (b) of Figure 2 show a clear trend of classroom radon concentrations increasing when the HVAC system was turned off, and decreasing when it was turned on. The school shown in panel (a) had classroom radon concentrations that remained above the EPA’s recommended action level for most of the school day, whereas the school depicted in panel (b) was able to maintain classroom radon concentrations below the EPA’s recommended action level for the majority of the school day.

## 4. Discussion

Our study found a limited number of classrooms with radon concentrations at or above the EPA’s recommended action level. We found that a small or large number of classrooms in a school was associated with higher classroom radon concentrations compared to schools with a medium number of classrooms. Additionally, the year that schools’ HVAC systems were installed was inversely related to having classroom radon concentrations at or above the EPA’s recommended action level. Number of classrooms and number of students in schools had a u-shape relationship with having classroom radon concentrations at or above 148 Bq/m^3^ (4.0 pCi/L). CRM tests indicated lower classroom radon concentrations when schools operated their HVAC systems.

Comparing studies of radon concentrations in schools across the U.S. or globally is problematic given that soil deposits of uranium-238 and its decay products, soil permeability, and building characteristics vary greatly by geographic region [18,19]. Nevertheless, a review of 63 studies across Europe, Asia, Africa, and North America found that average radon concentrations in schools ranged from a low of eight to a high of 245 Bq/m^3^ (0.22–6.62 pCi/L), with a GM of 36 Bq/m^3^ (0.97 pCi/L) [20]. Our GM classroom radon concentration (31.39 Bq/m^3^ (0.85 pCi/L)) across the 66 schools was lower than that reported by Zhukovsky et al. [20]. Our finding was also lower than that of a previous study in Sevier County, Utah, in which the average radon concentration across three schools (44 samples) was 37 Bq/m^3^ (1.0 pCi/L) [21]. However, concentrations averaged across or within schools are of limited public health use for protecting schoolchildren. Within a given school, radon concentrations can vary widely due to location of floor slab joints, full or partial basements under classrooms, the presence of crawl spaces and utility tunnels, and design and operation of HVAC systems [6]. Thus, without testing, it is not currently possible to predict which occupied rooms may exceed the EPA’s recommended action level. Based on the EPA’s National School Radon Survey, 19.3% of schools in the U.S. have at least one occupied room with radon concentrations that exceed 148 Bq/m^3^ (4.0 pCi/L). In our study, 20% of schools had at least one classroom that had a radon concentration at or above 148 Bq/m^3^ (4.0 pCi/L), which mirrored the EPA’s National School Radon Survey findings. Thus, our findings support the current EPA recommendation to test all ground-level rooms that are frequently occupied, occupied basement rooms, rooms adjacent to ground contact, and rooms that are above an unoccupied basement space [6].

Radon concentrations in school buildings may also differ significantly from radon concentrations in homes in the same community. The average radon concentration in Utah’s homes is 196.10 Bq/m^3^ (5.3 pCi/L) [8], compared to our GM classroom concentration of 31.39 Bq/m^3^ (0.85 pCi/L) for schools in our study. Our findings are similar to the study in Sevier County, Utah, in which average radon concentrations (37 Bq/m^3^ (1.0 pCi/L)) for all three schools were less than the average (181.30 Bq/m^3^ [4.9 pCi/L]) for homes in the same communities [21]. The spatial relationship between residential and school radon concentrations is a topic of ongoing study that may allow for the future development of mathematical models or refined geospatial maps that more accurately identify radon-prone areas [22,23]; however, there is currently a paucity of data in this area. Kitto [24] found no correlation between residential and school radon concentrations in New York State when comparing schools to homes in the same towns. Conversely, a study in Florida that used smaller geographic areas found schools located within 0.4 km (0.25 miles) of homes with radon concentrations greater than the EPA’s recommended action level were 2.8 times more likely to have radon concentrations greater than 148 Bq/m^3^ (4.0 pCi/L) [25]. A better understanding of the spatial relationship between residential and school radon concentrations may lead to refined, geographically-tailored testing methodologies or testing frequencies for schools in the future. However, future research regarding the spatial relationship between residential and school radon concentrations will need to account for differences in how schools are ventilated, built, and used compared to homes. In the interim, we recommend following current EPA guidelines for testing classrooms [6].

Exposure assessment studies of classroom characteristics and measured radon concentrations have been conducted largely in European schools and kindergartens. Assessments of radon concentrations in classrooms in Serbia and Portugal reported significant variability between classrooms located on ground floors compared to upper-floor classrooms [26,27]. Classroom radon concentrations in these studies varied significantly by the building’s method of construction (e.g., foundation vs. no foundation), types of materials used to build the schools (e.g., brick vs. concrete), and types of windows installed [28,29]. Although we did not evaluate associations between building materials and classroom radon concentrations in our study, we did find a positive, but nonsignificant, association between classroom radon concentrations and age of the school, which could be a proxy for differing construction methods and materials over time. We also reported a significant association between number of classrooms and classroom radon concentrations, with schools having 19–30 classrooms or 81–148 classrooms having higher GM classroom radon concentrations compared to schools with 31–80 classrooms. The number of classrooms in schools may vary by the size of the population that the school serves and its physical location. The distribution of the population and thus its schools in our study area were largely centered in a single urban area. Rural cities have the smallest populations and may have more schools with a smaller number of classrooms. Elementary schools also typically have a smaller number of classrooms relative to middle or junior high schools and high schools. However, the associations we reported between number of classrooms in the schools and classroom radon concentrations and having a classroom radon concentration at or above the EPA’s recommended action level were quite robust. Our analysis suggests that rurality or the school’s physical location, school grade, and other factors related to classroom size should be examined in more detail. In addition, temporal changes in school policies regarding the construction of schools and location of schools and the underlying radon emission potential of the soils on which schools were built are important factors that should be examined in future research.

In a multicontinent review, schools had 1.5 times the risk for radon concentrations above 300 Bq/m^3^ (8.1 pCi/L) compared to homes, which is three times as high as the World Health Organization’s (WHO) health standard of 100 Bq/m^3^ (2.7 pCi/L) and two times as high as the EPA’s recommended action level [20]. Ventilation appears to have a significant role in reducing school radon concentrations below health standards. In our study, the long-term presence of an HVAC system and individual HVAC system usage were associated with a significant reduction in the odds of having classroom radon concentrations at or above the EPA’s recommended action level. In particular, having the HVAC systems turned off during the hour when students are leaving school, an hour of high activity and use of open doorways, appears to play a critical role in reducing classroom radon concentrations below the EPA’s recommended action level. Radon is known to build up within classrooms on weekends and holidays [26]. A review of 63 studies from multiple continents suggests that low ventilation in schools during night hours may increase schools’ risk for radon concentrations above national and international health standards [20]. These conditions also occur if the HVAC system is turned off while the school is closed and vacant. A school’s student body size may affect usage of doors and windows, with large student body size associated with more usage and thus increased ventilation [26], but our study does not show a linear relationship between the odds of having classroom radon concentrations at or above the EPA’s recommended action level and increasing number of students.

The UDEQ expects the average radon concentrations measured by a CRM and short-term test kit to be within 37 Bq/m^3^ (1.0 pCi/L) of each other if placed side-by-side in the same location, for the same amount of time, etc. However, when the UDEQ finds elevated radon concentrations in a school, they like to verify those elevated concentrations with a CRM that measures radon concentrations in real-time and gives a measurement of radon concentrations every hour. This is critical because the UDEQ often finds that radon concentrations are below 148 Bq/m^3^ (4.0 pCi/L) while students and staff are in the school building, but the radon concentrations increase to at or above 148 Bq/m^3^ (4.0 pCi/L) when the school’s HVAC system is not running at normal capacity (e.g., after normal school hours or on weekends). If the UDEQ discovers this is the case, then they will fill out a report and leave it with the school. In addition, school maintenance staff can extend the amount of time the school’s HVAC system is on each school day so that radon concentrations are below 148 Bq/m^3^ (4.0 pCi/L) when students and staff are in the school. For our study, we recommend extending the amount of time the HVAC system is on each school day for the two schools for which we collected CRM data because both schools had classroom radon concentrations above the EPA’s recommended action level at the time teachers, administrators, and staff arrived in the school. In addition, the first school had classroom radon concentrations above the EPA’s recommended action level at the time students arrived in the school.

School-based radon research, appropriately, is heavily focused on children’s health, but should also consider occupational exposure to teachers, administrators, and staff. Next to the home, children and school employees spend more time at school than in any other microenvironment [30,31]. For grade school-age children (prekindergarten to 5th grade), who are more likely to spend their school day in a single classroom, this can be six hours per day or more, depending on participation in after school programs [30,32], and nine hours per day or more for teachers [33]. However, whereas students will likely only spend one school year in a given classroom for prekindergarten to 5th grade, or one period per day in a given classroom for their middle and high school years, teachers, administrators, and staff may spend several years to decades in the same room at the school. Considering that one’s radon dose is a function of airborne concentration, minute ventilation rate, and duration of time spent in the exposure environment, some school employees may receive significantly higher doses of radon compared to students. School maintenance and custodial workers may be particularly at risk because they are more likely to spend time in basements, tunnels, and other underground areas. To date there is little published data on workers’ radon exposures in schools, and few states currently require testing of schools [32]. To address these issues, we recommend additional research to understand occupational radon exposures to school employees, including teachers, administrators, staff, and maintenance/custodians. In addition, Gordon et al. [32] outline several recommendations that could be amended to serve as a framework for state-specific policies to protect school employees as well as students.

Radon mitigation in large buildings, such as schools, often requires varied and tailored strategies due to complexities encountered with building construction, gaps between subslab and occupied areas, pressure differentials created between supply and exhaust fans, design of HVAC systems, and the presence of complex utility systems [34,35]. Synnott et al. [36] categorized mitigation strategies as being active or passive. Active mitigation strategies require the use of mechanical systems, such as fans, while passive systems, once installed, do not require the use of mechanical systems. Studies show that active measures, such as subslab depressurization and use of dilution air ventilation, are generally preferred for occupied areas with high radon concentrations. Saum et al. [35] successfully mitigated high radon concentrations in four schools in Maryland using primarily subslab depressurization or subslab depressurization combined with passive measures such as sealing cracks between subslab areas and classrooms. Synnott et al. [36] found that both active and passive strategies resulted in significant decreases in radon concentrations in Irish schools, with active strategies (subslab depressurization and ventilating underfloor areas) showing the greatest reductions. Denman and Phillips [37] reduced radon concentrations by 89% across 20 schools using primarily active strategies. For schools with high classroom radon concentrations in our study, mitigation efforts should focus on the use of active and passive methods tailored to each affected area.

Although our project did not include a formal educational evaluation of radon testing as an experiential learning experience for university students, it aligns with recent calls for increased use of experiential and active learning in occupational and environmental health education [38]. To deploy short-term radon test kits in each school, we enlisted the help of 94 BYU undergraduate and graduate students. Most students were enrolled in environmental health courses, but we recruited students from other public health, environmental science, and elementary education courses at BYU via electronic fliers and presentations in these courses. To introduce the project to our environmental health students, we shared a 20 min electronic presentation about radon, its dangers, and our project. We then had students sign up for available time slots to deploy and retrieve short-term radon test kits at specific schools. We instructed students and other study personnel regarding how to correctly deploy and retrieve short-term radon test kits when they retrieved them from our office. After all short-term radon tests had been deployed and retrieved, we asked a subset of students some reflective questions about their participation in the project. Anecdotally, students liked applying what they learned in class to benefit the community, but they thought more flexibility was needed to fit radon testing into their busy schedules. Most students also thought an environmental sampling project should be included in environmental health courses in the future. Thus, radon testing may be an effective experiential learning experience for university students, but a more formal educational evaluation is needed in the future.

One strength of our study is that we included elementary, junior high or middle, and high schools, which may mean our results are applicable to all school grades. We also used the same short-term radon test kits that are used by local health departments and homeowners and the instructions we gave our students for deploying the short-term radon test kits followed the EPA’s guidelines [10]. Although we were limited in the number of classrooms for which we could conduct follow-up testing, we were able to conduct some follow-up testing using CRMs, which gave us an hourly measurement of radon concentrations in the tested classrooms and how HVAC system use affected those radon concentrations. We also investigated school characteristics that were associated with classroom radon concentrations and had a high response rate to our survey, as 65 (98%) schools answered at least some of our survey questions. Radon concentrations in schools were not spatially correlated, which supports the importance of the associations between school characteristics and classroom radon concentrations that we identified. We were able to involve 100 people, including 94 BYU undergraduate and graduate students, in testing schools for radon, which may have been a valuable experiential/service learning assignment for the students. Finally, our project was community-based and we shared the radon testing results with the school districts and schools that participated.

Our study had several limitations. Despite including test results for 2289 classrooms in 66 schools, we were likely underpowered to detect some significant differences (e.g., for school grade) and we were unable to conduct stepwise variable selection to develop multiple regression models. The Bonferroni method, which is a post-hoc adjustment to the significance level based on the number of statistical tests performed, may be too conservative (i.e., it will fail to reject the null hypothesis when it should be rejected) [39], but multiple comparison adjustment methods are not currently options in PROC NLMIXED [13]. In addition, PROC NLMIXED cannot be used to explore several possible working correlation matrices (e.g., first-order autoregressive) [13]. As discussed above, some of our results may have been confounded (e.g., associations between number of classrooms and classroom radon concentrations, classroom radon concentrations at or above the EPA’s recommended action level, and classroom radon concentrations above detection limits), but they also point to avenues for future research. Our use of a cross-sectional study design and short-term radon test kits may mean our results are not representative of long-term or annual classroom radon concentrations or trends [9]. However, to be most protective of students, teachers, administrators, and staff at each school and to provide a “worst-case” estimate of classroom radon concentrations, we followed the EPA’s guidelines and tested schools during the winter when radon concentrations are typically highest [9]. Future research could include long-term measurements to estimate long-term or annual classroom radon concentrations and trends. We also did not assess associations between radon concentrations in homes located within the vicinity of the school and school classroom radon concentrations. Instead, we focused primarily on school characteristics that are known to differ from homes based on their usage and construction; these differences may contribute to variation between radon concentrations found in schools and the homes surrounding them. Although we did not monitor outdoor radon concentrations, doing so was not necessary because outdoor concentrations are typically lower than indoor concentrations [9,40] and schools were not located near uranium ore waste sites or mines which typically increase radon concentrations [41]. Finally, our results may not apply to schools located in other areas because radon concentrations are known to vary geographically based on soil deposits of uranium-238 and its decay products, soil permeability, and building characteristics [18,19].

## 5. Conclusions

We found that the number of classrooms, the year HVAC systems were installed, number of students, and when HVAC systems were operated were associated with classroom radon concentrations and/or classroom radon concentrations at or above the EPA’s recommended action level. Most classrooms had radon concentrations below the EPA’s recommended action level; however, some classrooms had concentrations at or above 148 Bq/m^3^ (4.0 pCi/L). The results of our study and knowledge about radon-associated lung cancer indicate the importance of testing classrooms for radon. State radon programs should educate school administrators regarding the danger of radon concentrations and work with them to create a plan to test schools for radon, which is especially important for small and large schools. Schools that have classroom radon concentrations at or above the EPA’s recommended action level should implement mitigation strategies. Administrators should consider policies that require regular radon testing for schools. Schools may maintain radon concentrations below 148 Bq/m^3^ (4.0 pCi/L) by regularly operating their HVAC systems when students and staff are in the school, which could reduce the need to implement further mitigation strategies. Additional research about radon testing education for school administrators and staff and the impact of that education on actual radon testing of schools is needed.

## Figures and Tables

**Table 1:** Characteristics of schools for which radon testing was conducted, Utah, January–February 2019.

School Characteristic	N	%	Missing	Mean	SD	Min	Q1	Median	Q3	Max
Grade			0							
Elementary	46	70								
Junior high or middle	12	18								
High	8	12								
Age, years			2	29.09	21.66	3.00	13.00	23.00	38.75	120.00
Year HVAC system installed			4	1999	14.22	1954	1994	2002	2009	2017
Last year HVAC system maintained			4							
2012–2018	38	61								
2019	24	39								
Type of HVAC system			3							
Multizone system	45	71								
Single-zone or hydronic system	6	10								
Variable air volume system	6	10								
Combination of systems	6	10								
Time HVAC system turned on/started each day, HH:MM:SS			3	06:10:00	1:18:15	00:00:00	06:00:00	06:15:00	07:00:00	07:45:00
Time HVAC system turned off/stopped each day, HH:MM:SS			3	16:42:01	1:46:26	15:00:00	16:00:00	16:00:00	16:30:00	23:59:59
Total number of hours HVAC system on/running each day			2	10.51	2.90	7.75	9.00	9.90	10.50	24.00
HVAC system on/running during school hours			1							
No	0	0								
Yes	65	100								
Basement			1							
No	45	69								
Yes	20	31								
If basement, finished?			1							
No	14	74								
Yes	5	26								
Crawlspace or uncovered dirt floor			5							
No	47	77								
Yes	14	23								
Levels/Stories			2							
1	48	75								
2–3	16	25								
Classrooms			4	43.61	21.11	19.00	32.00	38.00	49.00	148.00
Students			4	978.42	604.00	182.00	642.00	819.00	1200.00	3300.00
Built using radon-resistant new construction			17							
No	34	69								
Yes	15	31								

Abbreviations: HVAC, heating, ventilation and air conditioning; Min, minimum; Max, maximum; Q1, first quartile; Q3, third quartile; SD, standard deviation.

**Table 2:** Radon testing results for 66 schools in Utah, January–February 2019.

	Classrooms					
Radon Variable	N	%	GM ^a^	95% CI ^a^	Min ^b^	Max ^b^
Below detection limits ^c^						
No	1491	65				
Yes	798	35				
Radon concentration, Bq/m^3^			31.39	27.16, 36.28	14.80	673.40
Below EPA’s recommended action level ^d^						
No	37	2				
Yes	2252	98				

Abbreviations: Bq/m^3^, becquerels per cubic meter of air; CI, confidence interval; EPA, U.S. Environmental Protection Agency; GM, geometric mean; Min, minimum; Max, maximum; pCi/L, picocuries per liter of air. ^a^ Estimated via a simple Tobit mixed regression model of the natural logarithm transformed values. ^b^ Minimum and maximum of samples that measured above detection limits. ^c^ Detection limits ranged from 11.1–55.5 Bq/m^3^ (0.3–1.5 pCi/L). ^d^ The EPA’s recommended action level is 148 Bq/m^3^ (4.0 pCi/L).

**Table 3:** Associations between school characteristics and classroom radon concentrations, Utah, January–February 2019.

	Radon Concentration, Bq/m^3^		
School Characteristic	GM ^a^	95% CI ^a^	*p*-Value ^a^
Grade			
Elementary	32.83	27.68, 38.94	
Junior high or middle	25.07	17.93, 35.06	
High	33.92	22.60, 50.92	0.34
Age, 10 years	1.02 ^b^	0.95, 1.09 ^b^	0.64
Year HVAC system installed			
1954–1970	37.15	20.79, 66.35	
1971–1985	35.52	22.91, 55.07	
1986–2000	28.61	21.57, 37.94	
2001–2017	31.60	25.88, 38.60	0.78
Last year HVAC system maintained			
2012	33.00	10.29, 105.86	
2018	32.89	27.11, 39.90	
2019	28.24	22.20, 35.93	0.61
Type of HVAC system			
Multi-zone system	30.91	25.97, 36.78	
Single-zone or hydronic system	31.87	19.77, 51.39	
Variable air volume system	41.02	25.48, 66.04	
Combination of systems	25.59	15.87, 41.28	0.57
Time HVAC system turned on/started each day, one hour	1.00 ^b^	0.88, 1.13 ^b^	0.97
Time HVAC system turned off/stopped each day, HH:MM:SS ^c^			
15:00:00–16:00:00	28.44	23.66, 34.20	
16:00:01–16:30:00	43.27	30.10, 62.19	
16:30:01–18:00:00	29.38	18.35, 47.02	
18:00:01–23:59:59	33.68	22.47, 50.48	0.23
Total number of hours HVAC system on/running each day, one hour	1.01 ^b^	0.96, 1.06 ^b^	0.76
Basement			
No	30.16	25.32, 35.91	
Yes	33.01	25.44, 42.85	0.57
If basement, finished?			
No	35.10	25.43, 48.44	
Yes	27.33	15.96, 46.80	0.41
Crawlspace or uncovered dirt floor			
No	30.28	25.46, 36.01	
Yes	33.18	24.19, 45.51	0.61
Levels/Stories			
1	31.26	26.37, 37.06	
2	29.89	21.24, 42.08	
3	33.48	18.65, 60.10	0.94
Classrooms			
19–30	42.60	30.95, 58.64	
31–40	29.42	24.24, 35.69	
41–50	23.02	15.84, 33.46	
51–60	22.98	15.36, 34.37	
61–70	24.22	11.52, 50.92	
71–80	57.12	20.13, 162.13	
81–148	58.59	32.03, 107.18	0.03 ^d^
Students, 100 students	1.00 ^b^	0.97, 1.02 ^b^	0.78
Built using radon resistant new construction			
No	31.28	26.01, 37.61	
Yes	35.31	26.78, 46.56	0.47
Mitigated for radon previously			
No	32.30	27.61, 37.80	
Yes	36.16	20.67, 63.28	0.70

Abbreviations: Bq/m^3^, becquerels per cubic meter of air; CI, confidence interval; GM, geometric mean; HVAC, heating, ventilation, and air conditioning. ^a^ Estimated via simple Tobit mixed regression models of the natural logarithm transformed values. ^b^ Exponentiated regression coefficient and 95% CI (i.e., GM radon concentration ratio for a specified change in the independent variable or exp(β) – 1 = percent change in GM radon concentration for a specified change in the independent variable). ^c^ Category boundaries set at quintiles of the distribution of time HVAC system turned off/stopped each day. ^d^ *p*-values for tests of pairwise differences among classroom categories were as follows: 19–30 vs. 31–40: 0.05; 19–30 vs. 41–50: 0.02; 19–30 vs. 51–60: 0.02; 19–30 vs. 61–70: 0.17; 19–30 vs. 71–80: 0.59; 19–30 vs. 81–148: 0.35; 31–40 vs. 41–50: 0.25; 31–40 vs. 51–60: 0.27; 31–40 vs. 61–70: 0.61; 31–40 vs. 71–80: 0.22; 31–40 vs. 81–148: 0.03; 41–50 vs. 51–60: 0.99; 41–50 vs. 61–70: 0.90; 41–50 vs. 71–80: 0.11; 41–50 vs. 81–148: 0.01; 51–60 vs. 61–70: 0.90; 51–60 vs. 71–80: 0.11; 51–60 vs. 81–148: 0.01; 61–70 vs. 71–80: 0.19; 61–70 vs. 81–148: 0.07; 71–80 vs. 81–148: 0.97.

**Table 4:** Associations between school characteristics and whether classroom radon concentrations were at or above the EPA’s recommended action level ^a^, Utah, January–February 2019.

		Classrooms ≥ EPA’s RAL		Classrooms < EPA’s RAL			
School Characteristic	Median	N	%	N	%	OR ^b^	95% CI ^b^
Grade							
Elementary		26	70	1330	59	1.00	Reference
Junior high, middle, or high		11	30	922	41	0.61	0.17, 2.19
Age, 10 years						1.20	0.95, 1.51
Missing		0		57			
Year HVAC system installed, 10 years						0.72	0.53, 0.99
Missing		0		136			
Last year HVAC system maintained							
2012–2018	2018	20	61	1368	66	1.00	Reference
2019	2019	13	39	710	34	1.25	0.24, 6.40
Missing		4		174			
Trend ^c, d^	Scaling factor: 1 year					1.25	0.24, 6.40
Type of HVAC system							
Multi-zone, variable air volume, or combination of systems		28	76	1979	93	1.00	Reference
Single-zone or hydronic system		9	24	150	7	4.24	0.56, 32.40
Missing		0		123			
Time HVAC system turned on/started each day, one hour						1.15	0.79, 1.67
Missing		0		127			
Time HVAC system turned off/stopped each day, HH:MM:SS							
15:00:00–16:00:00	16:00:00	9	24	1266	60	1.00	Reference
16:00:01–17:00:00	16:30:00	21	57	431	20	6.85	1.38, 34.14
17:00:01–23:59:59	19:15:00	7	19	428	20	2.30	0.54, 9.72
Missing		0		127			
Trend ^c, d^	Scaling factor: 1 h					1.09	0.81, 1.45
Total number of hours HVAC system on/running each day							
7.75–10	9.0	12	32	1328	61	1.00	Reference
>10–11	10.5	18	49	423	19	4.71	0.92, 24.09
>11–24	13.5	7	19	444	20	1.74	0.47, 6.47
Missing		0		57			
Trend ^c, d^	Scaling factor: 1 h					1.12	0.93, 1.35
Basement							
No		20	54	1495	67	1.00	Reference
Yes		17	46	724	33	1.76	0.42, 7.41
Missing		0		29			
Crawlspace or uncovered dirt floor							
No		21	58	1451	70	1.00	Reference
Yes		15	42	611	30	1.69	0.36, 7.92
Missing		1		186			
Levels/Stories							
1	1	25	68	1621	74	1.00	Reference
2	2	7	19	376	17	1.21	0.23, 6.37
3	3	5	14	198	9	1.64	0.39, 6.79
Missing		0		57			
Trend ^c, d^	Scaling factor: 1 level					1.26	0.58, 2.75
Classrooms							
19–30	26	22	61	259	12	19.40	4.59, 81.99
31–70	40	7	19	1599	76	1.00	Reference
71–148	95	7	19	245	12	6.53	2.04, 20.84
Missing		1		149			
Trend ^c, d^	Scaling factor: 10 classrooms					0.92	0.53, 1.60
Students ^e^							
182–650	530	23	64	484	23	9.32	2.23, 38.99
651–1500	1000	7	19	1373	65	1.00	Reference
1501–3300	2400	6	17	246	12	4.78	1.33, 17.24
Missing		1		149			
Trend ^c, d^	Scaling factor: 100 students					0.95	0.75, 1.19
Built using radon resistant new construction							
No		30	81	1171	68	1.00	Reference
Yes		7	19	546	32	0.50	0.10, 2.50
Missing		0		535			

Abbreviations: Bq/m^3^, becquerels per cubic meter of air; CI, confidence interval; EPA, U.S. Environmental Protection Agency; HVAC, heating, ventilation, and air conditioning; OR, odds ratio; pCi/L, picocuries per liter of air; RAL, recommended action level. ^a^ EPA’s recommended action level is 148 Bq/m^3^ (4.0 pCi/L). ^b^ Estimated via simple unconditional logistic regression models with generalized estimating equations and an independent working correlation matrix. ^c^ Used within-category medians that were calculated using all classrooms. ^d^ Scaled the OR and 95% CI using the scaling factor shown in the table. ^e^ Category boundaries set at quintiles of the distribution of students and then combined until all cells contained at least five students.

## Supplementary Materials

The following are available online at https://www.mdpi.com/1660-4601/17/16/5839/s1, Instructions for Radon Testing Schools, p. 2, Determinants of Radon Levels in Public Schools Questionnaire, p. 4, Table S1: Associations between school characteristics and whether classroom radon concentrations were above detection limits, Utah, January-February 2019.
