# Supplementary material for: Associations Between School Characteristics and Classroom Radon Concentrations in Utah’s Public Schools: A Project Completed by University Environmental Health Students
Source: Int J Environ Res Public Health. 2020 Aug 12;17(16):5839. doi: 10.3390/ijerph17165839 (PMC7460026; doi:10.3390/ijerph17165839)
Supplement: Supplementary file 1 [file ijerph-17-05839-s001.pdf]

International Journal of
Environmental Research and Public Health

Supplementary Material: Associations Between School Characteristics and Classroom Radon Concentrations in Utah's Public Schools: A Project Completed by University Environmental Health Students

Elizabeth A. Davis [1], Judy Y. Ou [2], Cheyenne Chausow [1], Marco A. Verdeja [1], Eleanor Divver [3], James D. Johnston [1] and John D. Beard [1,*]

[1] Department of Public Health, Brigham Young University, Provo, UT 84602, USA; eapowell@uci.edu (E.A.D.); c.chausow17@gmail.com (C.C.); marcoverdeja@hotmail.com (M.A.V.); james_johnston@byu.edu (J.D.J.)
[2] Cancer Control and Population Sciences, Huntsman Cancer Institute at the University of Utah School of Medicine, Salt Lake City, UT 84112, USA; Judy.Ou@hci.utah.edu
[3] Radon Program, Utah Department of Environmental Quality, Salt Lake City, UT 84116, USA; edivver@utah.gov
* Correspondence: john_beard@byu.edu; Tel.: +1-801-422-7491

INSTRUCTIONS FOR RADON TESTING SCHOOLS

School: <school name and address>

Contacts: <school administrator and contact information, school physical facilities manager or head custodian and contact information>

Please contact the school's administrator and physical facilities manager (if known) as soon as possible to remind them of the date and time you are coming to their school, to give them your names and contact information, and to ask them to notify their teachers that you will be placing radon test kits in their classrooms. An example email (or phone call) is

Subject: REMINDER: BYU students performing radon tests on <date>

Dear <contact name or names>,

This is a reminder that BYU students will be conducting radon tests at your school. We will be placing tests at <time> on <date> and picking up tests at <time> on <date>. We will check in at the office when we arrive and check out at the office when we leave. Please have someone available to help us access locked classrooms. Please also notify your teachers we will be placing radon tests that will be in their classrooms for two to three days. Please tell them they do not need to do anything for the tests other than make sure the tests are not moved or damaged. Please let us know if you have any questions. Our contact information is below. We look forward to meeting you soon and appreciate your willingness to have us test your school.

Thank you,

<student name, phone number, email address>
<student name, phone number, email address>

Supplies:
- Grocery bag
- Radon test kits
- Project information forms
- Questionnaire
- Pens
- Contact information for school (please see the top of the page)
- Envelopes (one manila, several white ones for the completed radon test kits)
- Testing procedures (please see below)

Day of test placement:
- Meet at the school at the designated time (you can also carpool there)
- Go to the office, check in, and ask for your contact (e.g., principal, physical facilities manager)
- Give the questionnaire to your contact (tell them they will be emailed a link to a Qualtrics version of the questionnaire). The questionnaire will take 15 minutes to complete and will help us make a better mitigation plan if any test shows levels of radon about the EPA limit.
- Walk with your contact through the school to the classrooms (only test classrooms on the ground floor or in the basement)

- One BYU student will place tests in the classrooms
  - Read the serial number to the other BYU student (so they can write it on the Device Placement Log)
  - Open the plastic bag and remove the test
  - Put the test paper side up in a place students cannot get to it
    - Test should be one foot from all interior walls
    - Test should be three feet from all exterior walls
    - Test should be at least 20 inches above the ground and no higher than seven feet above the ground, if possible (a filing cabinet is usually a good option)
    - Nothing should be within four inches of the test (you may need to move some things)
  - In the first classroom, place two tests side-by-side four inches from each other (one is a duplicate).
  - Conduct one "blank" test for the school (i.e., remove one test from the plastic bag, put it directly into the envelope, and then seal the envelope). Please remember to write the test serial kit # on the envelope before you seal it.
- One BYU student will fill out the project information forms
  - Fill out "Structure Type" on the Project Information Form
  - Fill out the Personnel Log
  - Complete the first five columns of the Device Placement Log (i.e., Test Serial Kit #, Test Location, Deployment Time, Deployment Date, Deployment Initials). You may also need to complete Comments.
  - Remember to include the duplicate test and "blank" test on the Device Placement Log
- Go to the office and check out

<u>Day of test pick-up:</u>

- Meet at the school at the designated time (you can also carpool there)
- Go to the office, check in, and ask for your contact (e.g., principal, physical facilities manager)
- Ask your contact if they can give you the completed questionnaire (you do not need to get the questionnaire back if they completed the Qualtrics version)
- Walk with your contact through the school to the classrooms
- One BYU student will pick up tests from the classrooms
  - Read the serial number to the other BYU student (i.e., so they can check that it matches the serial number on the Device Placement Log)
  - Write the serial number on the envelope
  - Put the test in the envelope
  - Seal the envelope
  - Remember that you will pick up two tests in the first classroom (one is a duplicate)
  - If you forgot to conduct a "blank" test when you placed tests, then please conduct a blank test now (i.e., remove one test from the plastic bag, put it directly into the envelope, and then seal the envelope). Please remember to write the test serial kit # on the envelope before you seal it.
- One BYU student will fill out the project information forms
  - Complete the last five columns of the Device Placement Log (i.e., Retrieval Time, Retrieval Date, Retrieval Initials, Closed, Comments). Comments should be completed if the test is missing, has been moved, is damaged, etc.
  - Remember to include the duplicate test and "blank" test on the Device Placement Log
- Go to the office and check out
- Bring all tests, forms, and supplies to Dr. Beard's office (2046 LSB) as soon as possible

Determinants of Radon Levels in Public Schools Questionnaire

Please answer the following questions. The questionnaire will take approximately 15 minutes to complete.

1. Name of school

____

2. Which school district does the school belong to?

____

3. What is the age of the school?

____

4. In what year was the school's heating, ventilation and air conditioning (HVAC) system installed?

____

5. When was the last year that the HVAC system was maintained?

____

6. What type of HVAC system does the school have? (Please select all that apply)

☐ Single-zone system
☐ Multi-zone system
☐ Variable air volume system
☐ Terminal reheat system
☐ Induction system
☐ Dual duct system
o Low-velocity dual duct system
o High-velocity dual duct system

☐ Other (please list):____

7. What time is the HVAC system turned on/started each day?

____

8. What time is the HVAC system turned off/stopped each day?

____

9. What is the total number of hours the HVAC system is on/running each day?

____

10. Is the HVAC system on/running during school hours?

☐ Yes, the HVAC system is on during school hours.
☐ No, the HVAC system is not on during school hours.

11. Is there a basement in the school?

☐ Yes, there is a basement in the school.
☐ No, there is not a basement in the school.

12. If there is a basement in the school, is it finished?

☐ Yes, the basement is finished.
☐ No, the basement is not finished.

13. Does the school have a crawlspace or an uncovered dirt floor?

☐ Yes, the school has a crawlspace or an uncovered dirt floor.
☐ No, the school does not have a crawlspace or an uncovered dirt floor.

14. How many levels/stories are there in the school?

____

15. How many classrooms are there in the school?

____

16. How many students attend the school?

____

17. Was the school built using radon resistant new construction?

☐ Yes, the school was built using radon resistant new construction.
☐ No, the school was not built using radon resistant new construction.

18. Has the school been mitigated for radon previously?

☐ Yes, the school been mitigated for radon previously.
☐ No, the school has not been mitigated for radon previously.

19. If the school was previously mitigated for radon, during what year was the school mitigated?

____

20. If the school was previously mitigated for radon, how was the school mitigated for radon/what method was used to mitigate the school? (Please select all that apply)

☐ Natural ventilation (opening windows/door weather permitting)
☐ Covering exposed dirt (e.g. in crawlspaces)
☐ HVAC system time increased
☐ Sealing cracks and openings
☐ Sub-slab depressurization system

☐ Other (please list):____

____

Thank you for completing the Determinants of Radon Levels in Public Schools Questionnaire and for your school's participation in this study!

Table S1. Associations between school characteristics and whether classroom radon concentrations were above detection limits[a], Utah, January-February 2019.

School Characteristic	Median	Classrooms > DL		Classrooms ≤ DL		OR[b]	95% CI[b]
		N	%	N	%		
Grade							
Elementary		907	61	449	56	1.00	Reference
Junior high or middle		287	19	192	24	0.66	0.28, 1.52
High		297	20	157	20	0.79	0.31, 2.03
Age, 10 years						1.02	0.88, 1.18
Missing		47		10			
Year HVAC system installed, 10 years						0.90	0.74, 1.10
Missing		81		55			
Last year HVAC system maintained							
2012	2012	36	3	15	2	1.18	0.77, 1.81
2018	2018	877	65	460	61	1.00	Reference
2019	2019	438	32	285	38	0.77	0.40, 1.49
Missing		140		38			
Trend[c, d]	Scaling factor: 1 year					0.90	0.71, 1.14
Type of HVAC system							
Multi-zone system		1,067	75	559	76	1.00	Reference
Single-zone or hydronic system		104	7	55	7	1.13	0.35, 3.68
Variable air volume system		181	13	39	5	1.93	0.42, 8.95
Combination of systems		80	6	81	11	0.61	0.27, 1.35
Missing		59		64			

School Characteristic	Median	Classrooms > DL N	%	Classrooms ≤ DL N	%	OR[b]	95% CI[b]
Time HVAC system turned on/started each day, HH:MM:SS							
00:00:00-05:00:00	05:00:00	185	13	27	4	3.43	1.31, 8.96
05:00:01-05:45:00	05:30:00	88	6	144	19	0.41	0.13, 1.23
05:45:01-06:30:00	06:00:00	684	49	380	50	1.00	Reference
06:30:01-07:15:00	07:00:00	319	23	160	21	1.13	0.57, 2.25
07:15:01-07:45:00	07:30:00	125	9	50	7	1.26	0.47, 3.36
Missing		90		37			
Trend[c, d]	Scaling factor: 1 hour					1.00	0.68, 1.46
Time HVAC system turned off/stopped each day, HH:MM:SS							
15:00:00	15:00:00	12	1	54	7	0.12	0.08, 0.18
15:00:01-15:45:00	15:30:00	139	10	68	9	1.11	0.55, 2.23
15:45:01-16:30:00	16:00:00	853	61	465	61	1.00	Reference
16:30:01-17:15:00	17:00:00	97	7	39	5	1.07	0.32, 3.61
17:15:01-18:00:00	18:00:00	20	1	21	3	0.50	0.33, 0.75
18:00:01-18:45:00	18:30:00	83	6	22	3	1.79	0.88, 3.65
18:45:01-19:30:00	19:15:00	73	5	40	5	0.51	0.05, 5.57
19:30:01-20:15:00	20:00:00	11	1	40	5	0.14	0.10, 0.22
20:15:01-23:59:59	22:00:00	113	8	12	2	3.05	0.96, 9.72
Missing		90		37			
Trend[c, d]	Scaling factor: 1 hour					1.05	0.85, 1.29
Total number of hours HVAC system on/running each day, one hour						1.04	0.96, 1.12
Missing		47		10			
Basement							
No		1,001	68	516	65	1.00	Reference
Yes		461	32	282	35	0.93	0.50, 1.73
Missing		29		0			

School Characteristic	Median	Classrooms > DL N	%	Classrooms ≤ DL N	%	OR[b]	95% CI[b]
If basement, finished?							
No		340	77	174	64	1.00	Reference
Yes		103	23	98	36	0.70	0.24, 2.05
Missing		18		10			
Crawlspace or uncovered dirt floor							
No		959	71	515	69	1.00	Reference
Yes		399	29	229	31	0.89	0.40, 1.98
Missing		133		54			
Levels/Stories							
1	1	1,047	73	599	76	1.00	Reference
2	2	247	17	136	17	1.00	0.41, 2.44
3	3	150	10	53	7	1.22	0.52, 2.84
Missing		47		10			
Trend[c, d]	Scaling factor: 1 level					1.06	0.67, 1.69
Classrooms							
19-24	23	67	5	19	2	2.08	0.61, 7.13
25-32	30	233	17	100	13	1.31	0.58, 2.95
33-40	38	487	35	288	38	1.00	Reference
41-48	45	93	7	103	14	0.58	0.19, 1.80
49-56	50	172	12	77	10	1.05	0.40, 2.74
57-64	60	92	7	105	14	0.51	0.11, 2.31
65-72	70	11	1	40	5	0.16	0.09, 0.27
73-80	78	71	5	7	1	5.80	3.42, 9.83
81-148	110	152	11	22	3	3.24	0.97, 10.82
Missing		113		37			
Trend[c, d]	Scaling factor: 10 classrooms					1.02	0.88, 1.18
Students, 100 students						0.98	0.93, 1.03
Missing		113		37			

School Characteristic	Median	Classrooms > DL		Classrooms ≤ DL			
		N	%	N	%	OR[b]	95% CI[b]
Built using radon resistant new construction							
No		758	64	443	77	1.00	Reference
Yes		419	36	134	23	1.83	0.90, 3.72
Missing		314		221			
Mitigated for radon previously							
No		1,184	93	598	96	1.00	Reference
Yes		91	7	23	4	2.21	0.98, 4.98
Missing		216		177			

Abbreviations: Bq/m^3, becquerels per cubic meter of air; CI, confidence interval; DL, detection limit; HVAC, heating, ventilation and air conditioning; OR, odds ratio; pCi/L, picocuries per liter of air.

[a] Detection limits ranged from 11.1-55.5 Bq/m^3 (0.3-1.5 pCi/L).

[b] Estimated via simple unconditional logistic regression models with generalized estimating equations and an exchangeable working correlation matrix.

[c] Used within-category medians that were calculated using all classrooms.

[d] Scaled the OR and 95% CI using the scaling factor shown in the table.

© 2020 by the authors. Licensee MDPI, Basel, Switzerland. This article is an open access article distributed under the terms and conditions of the Creative Commons Attribution (CC BY) license (http://creativecommons.org/licenses/by/4.0/).
